# Paeoniflorin and Albiflorin Attenuate Neuropathic Pain via MAPK Pathway in Chronic Constriction Injury Rats

**DOI:** 10.1155/2016/8082753

**Published:** 2016-06-26

**Authors:** Jianyu Zhou, Linyuan Wang, Jingxia Wang, Chun Wang, Zhihui Yang, Chenglong Wang, Yingli Zhu, Jianjun Zhang

**Affiliations:** ^1^Beijing University of Chinese Medicine, 11 Beisanhuandonglu, Chaoyang Qu, Beijing 100029, China; ^2^Chengde Medical University, Hebei, Chengde 067000, China; ^3^Department of Psychiatry, University of Florida, Gainesville, FL 32608, USA

## Abstract

Neuropathic pain remains as the most frequent cause of suffering and disability around the world. The isomers paeoniflorin (PF) and albiflorin (AF) are major constituents extracted from the roots of* Paeonia (P.) lactiflora* Pall. Neuroprotective effect of PF has been demonstrated in animal models of neuropathologies. However, only a few studies are related to the biological activities of AF and no report has been published on analgesic properties of AF about neuropathic pain to date. The aim of this study was to compare the effects of AF and PF against CCI-induced neuropathic pain in rat and explore the underlying mechanism. We had found that both PF and AF could inhibit the activation of p38 mitogen-activated protein kinase (p38 MAPK) pathway in spinal microglia and subsequent upregulated proinflammatory cytokines (interleukin-1*β* (IL-1*β*) and tumor necrosis factor-*α* (TNF-*α*)). AF further displayed remarkable effects on inhibiting the activation of astrocytes, suppressing the overelevated expression of phosphorylation of c-Jun N-terminal kinases (p-JNK) in astrocytes, and decreasing the content of chemokine CXCL1 in the spinal cord. These results suggest that both PF and AF are potential therapeutic agents for neuropathic pain, which merit further investigation.

## 1. Introduction

Neuropathic pain is one of the most ubiquitous diseases in the world [1]. Due to undesirable side effects of treatments and unknown mechanisms of pathological pain states [2, 3], the treatment of neuropathic pain is difficult with conventional methods. Accumulating evidence indicates that neuroinflammation may play a critical role in the initiation and maintenance of neuropathic pain, which is now considered to be a neuroimmune disorder [3–6]. Various studies showed that activation of the glial cells (microglia and astrocytes) contributes to central nervous system neuroinflammation and promotes central sensitization, as well as subsequent development and maintenance of neuropathic pain [7–9]. In addition, several studies have shown that inhibiting microglial and astrocytic activation has analgesic effects on neuropathy [10–12].

**Figure 1:**
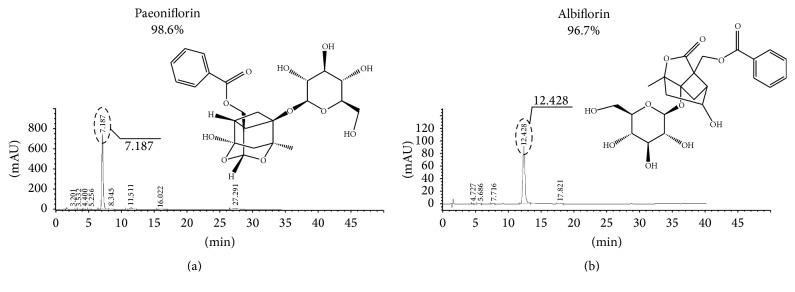
Chemical structures and HPLC chromatograms of paeoniflorin (a) and albiflorin (b).

Natural chemicals from food or herbs have been shown to be good for health and a valuable source for converting to leading drugs. Chinese herbs are important resources to develop safe and effective candidates for neuropathic pain therapy. Many plants have been proven effective for antagonizing chronic neuropathic pain [13–15].* P. alba* Radix, the dried roots of* P. lactiflora* Pallas or* P. veitchii* Lynch, is one of the traditional Chinese crude drugs. It has been widely used in traditional Chinese prescriptions to alleviate various disorders [16]. The isomers paeoniflorin (PF) and albiflorin (AF) are major constituents in* P. alba* Radix. PF (Figures 1(a) and 1(b)), a monoterpene glycoside, which has been reported to exhibit many pharmacological effects such as anti-inflammatory, antioxidant, and neuroprotective effects [17–20]. Our previous study has shown notable antinociceptive effect on antagonizing neuropathic pain in chronic constriction injury (CCI) rats and this discovery is further confirmed by the recently published research [21], which indicates that PF exert analgesic and hypnotic effects in partial sciatic nerve ligation mice.

Despite the fact that, compared to PF, only a few studies are related to the biological activities of AF and no report has been published on analgesic properties of AF about neuropathic pain to date, in the present study, we compared the effects of AF and PF against neuropathic pain and explored the underlying mechanism. To further explore their mechanism of antinociceptive activity, we focused on investigating the antineuroinflammation role of PF and AF using the CCI model. We examined the influence of PF and AF on spinal glial cell activation and further investigated the molecular pathways (MAPKs signaling pathways) in spinal cord glial cells related to CCI-induced neuropathic pain. Possible mechanisms by which PF and AF attenuated this pain behavior were explored.

## 2. Materials and Methods

### 2.1. Drugs

PF and AF were extracted from roots of* P. lactiflora* Pall and the preparative separation and purification were described previously [22]. The purity of PF is above 98% and AF is above 96% determined by high-performance liquid chromatographic (HPLC) assay (Figures 1(a) and 1(b)). PF and AF were dissolved in normal saline solution before use.

### 2.2. Animals

Seven-week-old Wistar rats (weight: 200–220 g) were obtained from SPF (specific pathogen-free) Animals Technology. Rats were housed on a 12-hour light and 12-hour dark schedule at a temperature of 23 ± 2°C and humidity of 60% ± 5% environment during the entire acclimatization (one week). The experiments were conducted according to the Guide for the Care and Use of Laboratory Animals (Ministry of Science and Technology of China, 2006). The use of the rat was reviewed and approved by the animal care committee in Beijing University of Chinese Medicine.

### 2.3. Chronic Constriction Injury (CCI) of the Sciatic Nerve

Animals were subjected to CCI as previously described by Bennett and Xie [23]. In brief, rats were anesthetized with chloral hydrate (300 mg/kg intraperitoneal injection) and the right sciatic nerve was exposed at a midthigh level. Proximal to the sciatic trifurcation, adhering tissue was removed from about 7 mm nerve and 4 ligatures (chromic catgut 4.0) were tied loosely at 1.0 mm intervals. Sham surgery was performed by exposing the right sciatic nerve without ligation.

### 2.4. Drug Treatment

Rats were randomly divided into 4 groups (14–16 rats in each group): Sham + vehicle (normal saline solution) group, CCI + vehicle group, CCI + PF group (50 mg/kg), and CCI + AF group (50 mg/kg). The optimal administration dosages of PF and AF were selected according to the results of the preliminary experiments. PF, AF, or vehicle was administered by intraperitoneal injections once a day for 15 days, starting on the first day after CCI.

### 2.5. Mechanical Withdrawal Threshold Testing

Mechanical allodynia was examined by assessing paw withdrawal threshold (PWT) in grams using calibrated von Frey filaments (North Coast Medical, Inc., Gilroy, CA) as described by Chaplan et al. [24]. Rats were placed in transparent Plexiglass cages on top of an elevated metal mesh floor and a series of von Frey filaments of logarithmically incremental stiffness were applied by Chaplan's up-down method at the central region of the plantar surface of right hind paw to identify the filament closest to the threshold of pain response. Each measurement was repeated three times at intervals of 15 minutes and the average force evoking reliable withdrawals was taken as threshold. Tests were performed 1 day before CCI surgery and 3, 7, 11, and 15 days after CCI surgery.

### 2.6. Thermal Withdrawal Latency Testing

Thermal hyperalgesia was measured using BME-410C thermal pain stimulator (Institute of Medical Biology, Chinese Academy Of Medical Sciences, Beijing, China) as described previously by Hargreaves et al. [25]. Rats were placed in transparent Plexiglass cages on top of an elevated glass platform and appropriate intensity radiant heat was applied from underneath the platform to the plantar surface of the hind paw until rats showed positive signs of pain. The time the rat took to lick or withdraw its paw was recorded and defined as the paw withdrawal latency (PWL). A cutoff time of 25 seconds was used to prevent tissue damage. Each measurement was repeated three times at intervals of 15 minutes and the average force evoking reliable withdrawals was taken as threshold. Tests were performed 1 day before CCI surgery and 3, 7, 11, and 15 days after CCI surgery.

### 2.7. Immunohistofluorescence Analysis

#### 2.7.1. Tissue Preparation and Immunofluorescence Labeling

On the 11th and 15th day after CCI surgery, 60 minutes after the last dose of drugs, rats were deeply anesthetized with chloral hydrate (350 mg/kg intraperitoneal). The L4-L5 spinal cord segments ipsilateral to the nerve injury were removed. The spinal cord was postfixed and paraffin was embedded and cut into 5 *μ*m sections. L4-L5 spinal cord sections were deparaffinized and subjected to heat mediated antigen retrieval using citric acid antigen repair buffer (pH 6.0). Sections were then incubated with tissue spontaneous fluorescence quenching agent (Goodbio technology Co., G1221) for 15 minutes at room temperature. Then sections were blocked using 3% BSA for 30 minutes at room temperature. Sections were then incubated with goat anti-Iba1 antibody (1 : 200, Abcam, ab5076), rabbit anti-GFAP (1 : 100, Goodbio technology Co., GB13095) or rabbit anti-phospho-p-ERK (1 : 100, Goodbio technology Co., GB13004) or mouse anti-phospho-p-JNK (1 : 100, Goodbio technology Co., GB13019), and rabbit anti-phospho-p38 MAPK (1 : 200, Cell Signaling Technology, CST4631). After extensive washing in PBS, the sections were incubated with biotinylated secondary antibodies of appropriate species (1 : 200 in PBS, Goodbio technology Co.) at room temperature for 50 minutes. For double immunofluorescence, sections were incubated with a mixture of primary antibodies followed by a mixture of fluorescein isothiocyanate or 488-conjugated secondary antibodies and Cy3-conjugated secondary antibodies. Spinal cord sections were washed with PBS, mounted in Antifade Mounting Medium (Beyotime Institute of Biotechnology, P0126), and scanned using a fluorescence microscope (Nikon, Eclipse CI). Photographs were taken with a digital camera system (Nikon, DS-U3) with the same camera parameters.

#### 2.7.2. Quantification of Immunohistochemical Labeling

To quantify immunofluorescence staining, 4 successive sections of L4-L5 spinal cord per animal were measured. The Iba-1 and GFAP immunoreactivity were quantified using NIH Image J software in a completely blind manner. Briefly, the images were converted to gray scale and black-white reversing processing pattern with NIH Image J. Using the same software, we analyzed the mean density level in a defined area of the spinal cord dorsal horn, which reflects the intensity of immunostaining. Immunohistochemical data were expressed as the fold change compared with data from Sham animals, which were considered to be 1.

### 2.8. Enzyme-Linked Immunosorbent Assay

On the 15th day after CCI surgery, 60 minutes after the last dose of drugs administration, rats were deeply anesthetized with chloral hydrate (350 mg/kg intraperitoneal). The L4-L5 spinal cords ipsilateral to the nerve injury were removed, frozen in liquid nitrogen, and stored at −80°C until further processing. Frozen spinal cords were homogenized in cold phosphate-buffered solution. After centrifugation at 10 000 ×g for 15 minutes, supernatant was used for enzyme-linked immunosorbent assay (ELISA). The contents of cytokines (TNF-*α*, IL-1*β*, and IL-6) and chemokine CXCL1 were measured by ELISA kits (Cusabio Biotech Co., Ltd., Wuhan, China) according to the manufacturer's instructions.

### 2.9. Western Blot Analysis

The L4-L5 spinal cords ipsilateral to the nerve injury were removed 60 minutes after the last dose of drugs administration on the 15th day after CCI. The tissue was homogenized and subsequently lysed in ice-cold lysis buffer containing 1 mM phenylmethylsulfonyl fluoride and a protease inhibitor mixture. The sample was subjected to centrifugation at 12 000 ×g for 10 minutes at 4°C. The supernatant was stored at −80°C until further processing. An equal amount of protein sample was loaded and separated by sodium dodecyl sulfate- (SDS-) polyacrylamide gel electrophoresis (PAGE). The resolved proteins were transferred onto nitrocellulose membranes (Millipore Corporation, Billerica, MA). The membranes were then blocked in 5% nonfat milk for 2 hours at room temperature and incubated overnight at 4°C with rabbit anti-phospho-p38 MAPK (1 : 1000, Cell Signaling Technology, CST4631), rabbit anti-p38 MAPK (1 : 1000, Cell Signaling Technology, cat. number CST8690), rabbit anti-p-ERK (1 : 1000, Cell Signaling Technology, CST9102), rabbit anti-phospho-p-ERK (1 : 1000, Cell Signaling Technology, CST9101), rabbit anti-p-JNK (1 : 1000, Cell Signaling Technology, CST9252), and rabbit anti-phospho-p-JNK (1 : 1000, Cell Signaling Technology, CST9255) individually. The blots were then incubated with goat anti-rabbit secondary antibodies (1 : 5000, Jackson ImmunoResearch Laboratories, USA) conjugated with horseradish peroxidase (1 : 500, ZSGB-BIO, Beijing, China) for 1 hour at room temperature. The protein bands were visualized with chemiluminescence reagent (ECL, Engreen Biosystem, Beijing, China). Western blot densitometry analysis of signal intensity was performed using NIH Image J software.

### 2.10. Statistical Analysis

The effect of each treatment was analyzed by software SPSS 17.0. All data were expressed as means ± standard error of the mean (SEM). Statistical analysis was performed using one-way analysis of variance, followed by the Least-Significant Difference (LSD)* post hoc* test or Dunnett T3 test for comparison of multiple groups. Statistical significance was considered at *P* < 0.05.

## 3. Result

### 3.1. Effects of PF and AF on the PWT and PWL in CCI Rats

**Figure 2:**
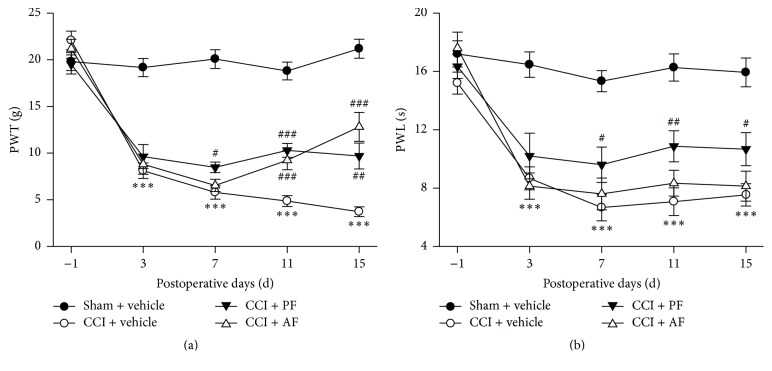
Effects of PF and AF on paw withdrawal threshold (PWT) and paw withdrawal latency (PWL) in CCI rats. (a) PWT was determined by von Frey filament test and (b) PWL was measured by thermal pain stimulator. Data were expressed as mean ± SE and *n* = 15–17 rats/group. ^*∗∗∗*^ *P* < 0.001 compared with the Sham group; ^#^ *P* < 0.05, ^##^ *P* < 0.01, and ^###^ *P* < 0.001 compared with the CCI group.

The effects of PF and AF on PWT (g) and PWL (s) in CCI rats are shown in Figures 2(a) and 2(b). Before CCI surgery, there was no significant difference of the baseline of PWL and PWT among all groups. PWT and PWL in CCI groups decreased markedly at 3rd, 7th, 11th, and 15th day after surgery compared with that in the Sham group (*P* < 0.001), indicating that CCI induced an obvious and long-lasting thermal hyperalgesia and mechanical allodynia. After the administration of PF, both PWL and PWT were increased strikingly in rats compared with that in the CCI group at 7th, 11th, and 15th day after surgery. However, AF treatment significantly increased the PWT on the 11th and 15th day after surgery, but AF was useless against the decreased PWL induced by CCI.

### 3.2. Effects of PF and AF on the Activation of Astrocytes and Microglia in CCI Rats

**Figure 3:**
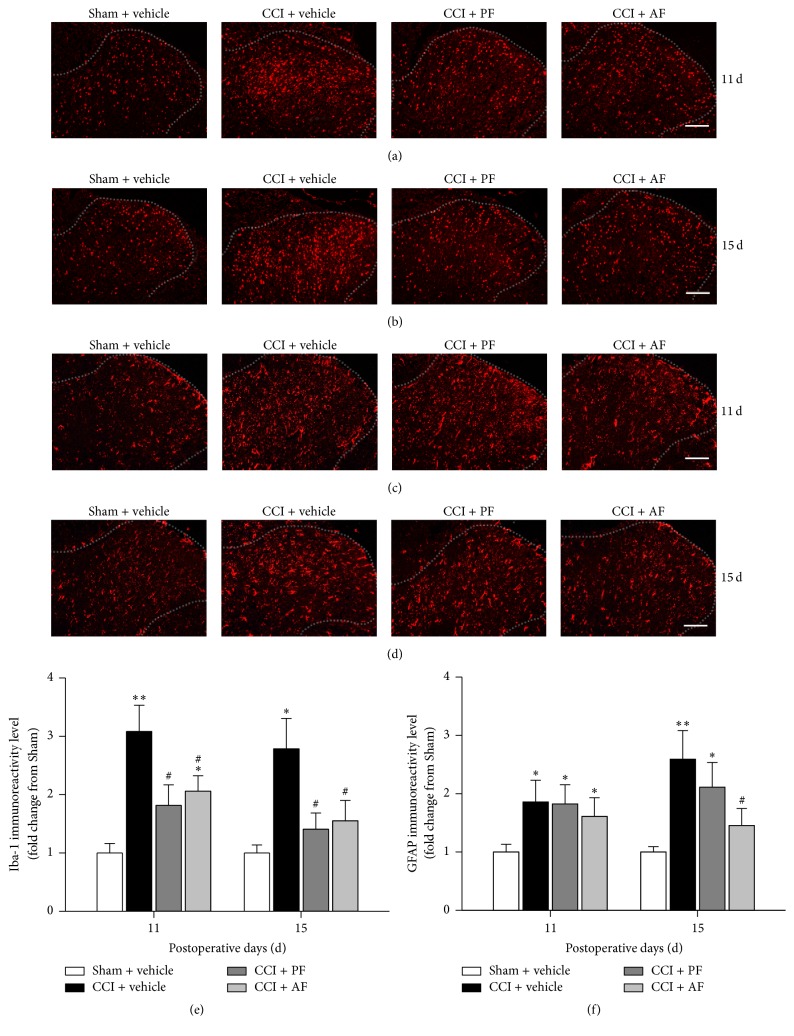
Effects of PF and AF on activation of microglia and astrocytes in the spinal cord dorsal horn of CCI rats. Spinal microglia and astrocytes activation were observed by immunohistochemistry using microglia marker Iba-1 and astrocyte marker GFAP, respectively. ((a)–(d)) Representative photographs of spinal cord dorsal horn sections of immunofluorescent labeling Iba-1 at 11th (a) and 15th (b) day after CCI surgery and immunofluorescent labeling GFAP at 11th (c) and 15th (d) day after CCI surgery. (e) Quantification of Iba-1 immunoreactivity. (f) Quantification of GFAP immunoreactivity. Results are presented as a fold of Sham control. Data were expressed as mean ± SE and *n* = 5-6 rats/group. ^*∗*^ *P* < 0.05 and ^*∗∗*^ *P* < 0.01 compared with the Sham group. ^#^ *P* < 0.05 compared with the CCI group.

To explore the possible mechanisms of PF and AF protective effects on neuropathic pain in rats, activation of astrocytes and microglia was monitored. Spinal cord sections were stained with antibodies specific to markers of microglia (Iba-1) and astrocytes (GFAP). As shown in Figures 3(a), 3(b), and 3(e), compared with the Sham group, CCI rats showed increased activation of microglia (Iba-1) at 11th and 15th day after CCI. Activation of microglia was decreased by administration of PF and AF at the two time points. Meanwhile, astrocytes (GFAP) were activated in CCI rats and such induction was greatly reduced by AF treatment, but not PF treatment (Figures 3(c), 3(d), and 3(f)).

### 3.3. Effects of PF and AF on the Levels of IL-1*β*, IL-6, TNF-*α*, and CXCL1 in Spinal Cord of CCI Rats

**Figure 4:**
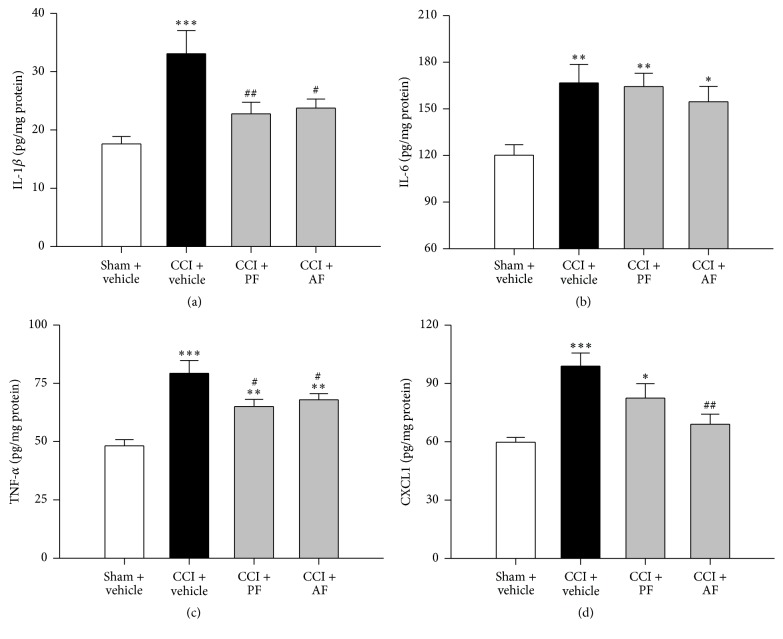
Effects of PF and AF on the elevated proinflammatory cytokines (TNF-*α*, IL-*β*, and IL-6) and chemokine CXCL1 levels in the spinal card dorsal horn of CCI rats. After treatment with PF for 15 days following CCI, the levels of IL-*β* (a), IL-6 (b), TNF-*α* (c), and CXCL1 (d) were measured by enzyme-linked immunosorbent assay; scale bar = 100 *μ*m. Data were expressed as mean ± SE and *n* = 5 rats/group. ^*∗*^ *P* < 0.05, ^*∗∗*^ *P* < 0.01, and ^*∗∗∗*^ *P* < 0.001 compared with the Sham group. ^#^ *P* < 0.05 and ^##^ *P* < 0.01 compared with the CCI group.

To investigate the effects of the PF and AF on CCI-induced neuroinflammation, the contents of proinflammatory cytokine levels (IL-1*β*, IL-6, and TNF-*α*) and chemokine CXCL1 in spinal cord samples were determined. As shown in Figures 4(a), 4(b), 4(c), and 4(d), compared with the Sham group, the levels of IL-1*β*, IL-6 TNF-*α*, and CXCL1 were markedly increased in the spinal cord of rats at 15th day after CCI. Compared to the CCI group, the PF and AF treatment significantly decreased the IL-1*β* and TNF-*α* levels. Furthermore, administration of AF remarkably decreased the CXCL1 level individually. However, neither PF nor AF exerted any effect on the CCI-induced upregulation of IL-6.

### 3.4. Effects of PF and AF on CCI-Augmented MAPKs Proteins

**Figure 5:**
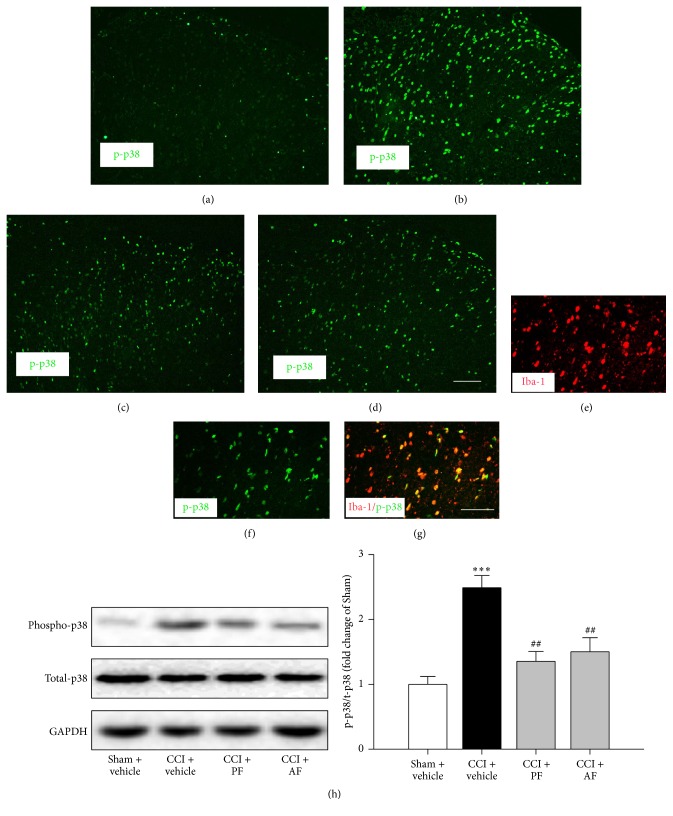
Effects of PF and AF on CCI-Augmented p-p38 MAPK protein in the spinal cord dorsal horn at the 15th day after CCI surgery. Immunofluorescent labeling p-p38 in Sham + vehicle (a), CCI + vehicle (b), CCI + PF (c), and CCI + AF (d). Scale bar = 100 *μ*m. ((e)–(g)) Double-immunofluorescent labeling p-p38/Iba-1. Scale bar = 50 *μ*m. (h) Quantification of p-p38 levels in the dorsal horn. The Western blot results are presented as a fold of Sham control, data were expressed as mean ± SE, and *n* = 5 rats/group. ^*∗∗∗*^ *P* < 0.001 compared with Sham + vehicle group and ^##^ *P* < 0.001 compared with CCI + vehicle group.

**Figure 6:**
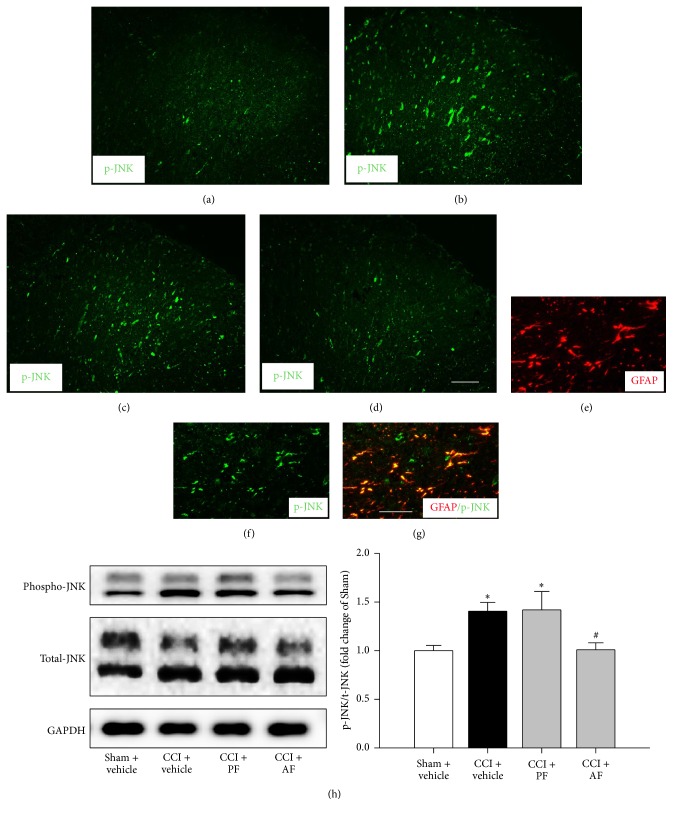
Effects of PF and AF on CCI-Augmented p-JNK protein in the spinal cord dorsal horn at the 15th day after CCI surgery. Immunofluorescent labeling p-JNK in Sham + vehicle (a), CCI + vehicle (b), CCI + PF (c), and CCI + AF (d). Scale bar = 100 *μ*m. ((e)–(g)) Double-immunofluorescent labeling p-JNK/GFAP. Scale bar = 50 *μ*m. (h) Quantification of p-JNK levels in the dorsal horn. The Western blot results are presented as a fold of Sham control, data were expressed as mean ± SE, and *n* = 5 rats/group. ^*∗*^ *P* < 0.05 compared with Sham + vehicle group and ^#^ *P* < 0.05 compared with CCI + vehicle group.

To further explore the mechanisms of PF and AF, we investigated the MAPKs proteins expression in spinal cord. The expression levels of p-p38 (Figure 5), p-JNK (Figure 6), and phosphorylation of extracellular signal-related kinases (p-ERK) (data not shown) were increased in spinal cord of CCI rats at 15th day after surgery. Both the PF and AF treatment decreased the elevated level of p-p38 compared with that in the CCI group (Figures 5(a), 5(b), 5(c), and 5(d)). At the same time point, AF-treated CCI group, but not PF, showed significant effect on elevated level of p-JNK (Figures 6(a), 6(b), 6(c), and 6(d)). However, neither the administration of PF nor AF took effect on the elevated p-ERK (data not shown) expression induced by CCI. These results were further confirmed by Western blotting (Figures 5(h) and 6(h)). Double immunostaining of p-p38/Iba-1 (specific markers of microglia) and CXCL1/GFAP (specific markers of astrocytes) in the dorsal horn of CCI 15-day animals indicated that p-p38 and p-JNK were colocalized in spinal cord microglia and astrocytes, respectively (Figures 5(e), 5(f), 5(g), 6(e), 6(f), and 6(g)). The above results demonstrated that both PF and AF inhibited the p38 MAPK pathway which was activated specifically in spinal cord microglia of CCI rats at 15th day after surgery. Meanwhile, AF, but not PF, suppressed the CCI-Augmented p-JNK expressed principally in spinal cord astrocytes. However, neither PF nor AF exerted any effect on the CCI-induced upregulation of p-ERK (data not shown).

## 4. Discussion

**Figure 7:**
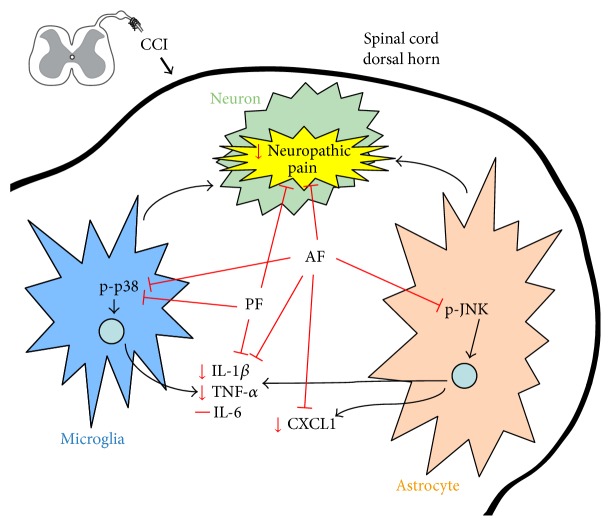
Schematic shows how PF and AF in the spinal cord dorsal horn regulate neuroinflammation-induced neuropathic pain.

The present study demonstrated for the first time that AF could alleviate the neuropathic pain (mechanical hyperalgesia) induced by CCI in rats and it could decrease the levels of proinflammatory cytokines (TNF-*α* and IL-1*β*) in spinal cord. In addition, PF and AF both inhibited the activation of microglia and reduced the activated p38 MAPK signaling pathway induced by CCI. Moreover, AF, but not PF, further displayed remarkable effects on inhibiting the activation of astrocytes, suppressing the intracellular overelevated expression of p-JNK in astrocytes and decreasing the content of chemokine CXCL1 in the spinal cord. Thus differential mechanisms may be involved in alleviating neuropathic pain of the two isomers (Figure 7).

After peripheral nerve injury, sensitized primary afferent terminals release nociceptive neurotransmitters and mediators which activate spinal microglia and astrocytes. Activated microglia and astrocytes contribute to neuroinflammation and accelerate facilitatory pain transmission, as well as subsequent development and maintenance of neuropathic pain [7–9]. To explore the possible mechanisms of PF and AF, activation of glial cells (astrocytes and microglia) was monitored. Our results showed that the administration of PF and AF could inhibit the activation of microglia induced by CCI. Moreover the administration of AF, but not PF, further suppressed the activation of astroglia at 15th day after surgery. The extra inhibiting effect on the activated astroglia may be the cause that AF exerted more robust analgesic effect (without statistically significant differences) after 11-day treatment.

MAPKs are crucial molecules in cell signaling, which consists of p38 MAPK, extracellular signal-related kinases (ERK1/2), and JNK1/2 [26]. Nerve injury or spinal cord injury induces a marked activation of MAPKs in glial cells in the spinal cord [27–30]. Subsequent studies have demonstrated that inhibition of JNK, p38, and ERK has been shown to effectively attenuate nerve injury induced neuropathic pain in various animal models [31]. Moreover, ERK plays an important role in neuronal plasticity in the adult [32], while p38 and JNK play essential roles in regulating inflammatory responses and neurodegeneration [33–35]. In our present study, PF treatment inhibited the activation of p38, while AF inhibited the activation of p38 and JNK, which indicates the two isomers may primarily exert analgesic effect via suppressing neuroinflammatory responses, but not influencing neuronal plasticity.

Emerging evidence indicates that nerve injury results in the activation of p38 MAPK in spinal cord and p38 MAPK regulates the production of the proinflammatory cytokines, promoting the development of neuropathic pain [36–38]. More recent studies report that p38 MAPK plays a critical role in microglial signaling under neuropathic pain conditions and represents a valuable therapeutic target for neuropathic pain [39, 40]. Furthermore, a growing body of literature indicates that proinflammatory cytokine is a critical factor in the initiation and maintenance of hyperalgesia in animal models of neuropathic pain [5]. The proinflammatory cytokine-mediated process during neuroinflammation can be induced by the nerve injury [6]. The CCI model induces upregulation of proinflammatory cytokines, such as IL-1*β*, IL-6, and TNF-*α* in the spinal cord [41, 42]. The increased proinflammatory cytokines in the spinal cord promote the transduction of detrimental signals by increasing excitatory synaptic transmission, decreasing inhibitory synaptic transmission [43]. The present study indicated that both PF and AF treatment prevented the CCI-induced upregulation of p-p38 proteins level as measured at 15th day after nerve injury. Moreover, this result has the same trend with the effect of the two isomers on reducing the increased levels of proinflammatory cytokines (IL-1 and TNF-*α*) (Figures 5(a), 5(b), 5(c), 5(d), 5(h), 4(a), and 4(b)). Furthermore, double staining shows that the p-p38 is colocalized with spinal cord microglia of CCI rats (Figures 5(e), 5(f), and 5(g)). Taken together, the above results suggest both PF and AF could inhibit the activation of p38 MAPK pathway in spinal microglia and subsequent upregulated proinflammatory cytokines, which may be beneficial for improving neuropathic pain.

Previously studies have demonstrated that JNK activation plays important roles in the pathological process of chronic pain [34]. Inhibition of JNK activation exerts antinociceptive effect in chronic pain animal models [44, 45]. Increasing evidence suggests an important role of chemokines in the genesis of neuropathic pain via regulating neuronal-glial interactions [46–49]. CXCL1 belongs to the chemokine family and plays an important role in the maintenance of central sensitization and neuropathic pain. Nerve injury induces persistent CXCL1 upregulation in spinal astrocytes, which is dependent on the JNK pathway and spinal inhibition of CXCL1 partly reversed nerve injury induced pain hypersensitivity [48, 49]. In our present study, AF decreased the upregulation of p-JNK proteins level and the CXCL1 level in the spinal cord. Moreover, double staining shows that the p-JNK is colocalized with spinal cord astrocytes (Figures 6(e), 6(f), and 6(g)). Taken together, the above results suggest AF, but not PF, could inhibit the activation of p-JNK pathway in spinal astrocytes and subsequent upregulated CXCL1.

Nerve injury induces p38 MAPK in spinal microglia especially in the acute stages [39], while astrocytic glial signaling in the spinal cord plays an important role in late phase neuropathic pain [50]. In our present study, AF not only inhibited the activation of p38 MAPK in spinal cord microglia, but also suppressed the activation of astroglia and JNK pathway, which indicates AF may have the analgesic effect both in acute and in late stages of neuropathic pain.

It should be noted that we fail to explain the pharmacodynamic action contrast between PF and AF on thermal hyperalgesia in the present study. Moreover, the duration of experiment may be not enough to conduct further research. Thus further studies will be summarized in our next study. Despite its preliminary character, this study can clearly indicate that both PF and AF have significant antinociceptive effect on CCI rat via inhibiting neuroinflammation in spinal cord, which might provide more documents on screening potential therapeutic agents for neuropathic pain.

## 5. Conclusions

Our results demonstrate that both PF and AF have significant antinociceptive effect on CCI rat via inhibiting neuroinflammation in spinal cord; meanwhile differential mechanisms may be involved in alleviating neuropathic pain of the two isomers. These results suggest that both PF and AF are potential therapeutic agents for neuropathic pain, which merit further investigation.
